# Development and validation of a novel prognostic signature in gastric adenocarcinoma

**DOI:** 10.18632/aging.104161

**Published:** 2020-11-08

**Authors:** Rui Mao, Zheng Wang, Yuanchuan Zhang, YuanYuan Chen, Qian Liu, Tongtong Zhang, Yanjun Liu

**Affiliations:** 1 Affiliated Hospital of Southwest Jiaotong University, Chengdu, 610036, China; 2 Department of Colorectal Surgery, National Cancer Center/National Clinical Research Center for Cancer/Cancer Hospital, Chinese Academy of Medical Sciences and Peking Union Medical College, Beijing, 100021, China; 3 The Center of Gastrointestinal and Minimally Invasive Surgery, The Third People’s Hospital of Chengdu, Chengdu, 610031, China; 4 Department of Pathology, The Third People’s Hospital of Chengdu, Chengdu, 610031, China; 5 Medical Research Center, The Third People’s Hospital of Chengdu, The Affiliated Hospital of Southwest Jiaotong University, The Second Chengdu Hospital Affiliated to Chongqing Medical University, Chengdu 610031, Sichuan, China

**Keywords:** gastric adenocarcinoma, qRT-PCR, competing endogenous RNA network, weighted gene coexpression network analysis

## Abstract

Competing endogenous RNA networks have attracted increasing attention in gastric adenocarcinoma (GA). The current study aimed to explore ceRNA-based prognostic biomarkers for GA. RNA expression profiles were downloaded from TCGA and GEO databases. A ceRNA network was constructed based on the most relevant modules in the weighted gene coexpression network analysis. Kaplan-Meier (KM) survival analysis revealed prognosis-related RNAs, which were subjected to the multivariate Cox regression analysis. The predictive accuracy and discriminative ability of the signature were determined by KM analyses, receiver operating characteristic curves and area under the curve values. Ultimately, we constructed a ceRNA network consisting of 55 lncRNAs, 17 miRNAs and 73 mRNAs. Survival analyses revealed 3 lncRNAs (LINC01106, FOXD2-AS1, and AC103702.2) and 3 mRNAs (CCDC34, ORC6, and SOX4) as crucial prognostic factors; these factors were then used to construct a survival specific ceRNA network. Patients with high risk scores exhibited significantly worse overall survival than patients with low risk scores, and the AUC for 5*-*year survival was 0.801. A total of 112 GA specimens and the GSE84437 dataset were used to successfully validate the robustness of our signature by qRT-PCR. In summary, we developed a prognostic signature for GA, that shows better accuracy than the traditional TNM pathological staging system.

## INTRODUCTION

Despite technological advances in diagnosis and treatment, stomach cancer remains the fourth most common cancer with the second highest mortality rate [1]. Although some prognostic factors, such as genes and the tumor microenvironment, have been evaluated, the exact mechanisms involved remain unclear [2–4]. The development of a prognostic marker that can accurately predict clinical results will better serve as a guide in the clinic. Long noncoding RNAs (lncRNAs) are more than 200 bp in length and lack coding ability. Several studies suggest that lncRNAs participate in the regulation of tumor progression and tumor biological behavior by interacting with microRNAs (miRNAs) or messenger RNAs (mRNAs). LncRNAs containing miRNA response elements can compete with miRNA target genes and regulate their expression by reducing free functional miRNAs. This kind of lncRNA is called a competitive endogenous RNA (ceRNA) [5–8]. This hypothesis has attracted increasing attention [9]. For example, Chen et al. explored whether the lncRNA PVT1 promotes tumor progression by regulating the miR-143/HK2 axis in gallbladder cancer [10]. Additionally, Wang et al. found that a novel lncRNA, MCM3AP-AS1, promotes the growth of hepatocellular carcinoma by targeting the miR-194-5p/FOXA1 axis [11]. Gastric cancer development in attributed to an imbalance between protein-coding and noncoding genes, and the regulatory mechanism of ceRNAs may be involved in this pathogenic process.

To identify ceRNAs associated with prognosis in GA and guide clinical applications, we integrated RNA-seq data from TCGA and GEO datasets and 112 GA specimens to establish a signature based on ceRNAs. Functional enrichment analysis and gene set enrichment analysis (GSEA) were performed to predict the potential functions of the genes in the ceRNA network.

## RESULTS

### Data acquisition and preprocessing

We collected gene matrix and clinical information from a TCGA data set, including 376 tumor tissues and 32 normal tissues. We excluded patients with a survival time of zero or incomplete clinical and pathological data. After screening, 349 tumor and 32 normal samples remained. After annotation, we obtained 19,754 mRNAs and 14,848 LncRNAs. The GSE84437 dataset was used as a validation cohort and contains 431 GA patients with a nonzero survival time.

Moreover, from June 2017 to August 2019, a total of 112 frozen, surgically resected tumor tissues were obtained from patients with a pathological diagnosis of GA at the Department of Pathology, Chengdu Third People's Hospital. The clinicopathological data of the TCGA and real-time quantitative PCR (qRT-PCR) datasets are presented in Table 1.

**Table 1:** The clinicopathological data of TCGA and qRT-PCR datasets.

**AJCC**	**TCGA dataset (n=349)**		**qRT-PCR dataset (n=112)**	
**Vital status**	Alive	Dead	Alive	Dead
**T Stage**	207	142	67	45
T1a	6	4	9	1
T1b	2	7	4	1
T2	44	30	10	4
T3	113	49	2	0
T4a	30	36	29	19
T4b	12	16	13	20
**N Stage**				
N0	64	44	36	11
N1	60	35	21	16
N2	42	33	9	15
N3a	37	24	1	3
N3b	4	6	0	0
**M Stage**				
M0	188	124	67	39
M1	19	18	0	6
**Pathological stage**				
IA	6	8	13	2
IB	20	16	8	3
IIA	43	22	3	1
IIB	47	28	11	4
IIIA	36	22	22	15
IIIB	26	18	10	13
IIIC	10	10	0	1
IV	19	18	0	6

Abbreviations: AJCC: American Joint Committee on Cancer.

### Differential expression analysis

After obtaining the expression data, we identified differentially expressed genes among tumorous and normal GA samples in the TCGA dataset using the software package edgeR and selected genes that were at least 2-fold higher in GA samples than in normal samples (Poisson model FDR < 0.05). Ultimately, we obtained 4721 differentially expressed mRNAs/lncRNAs (Supplementary Figure 1A, 1B). For miRNAs, if their expression deviated by more than 1.2 among these samples, they were subjected to WGCNA. Ultimately, we obtained 486 miRNAs for subsequent analyses.

### WGCNA

**Figure 1:**
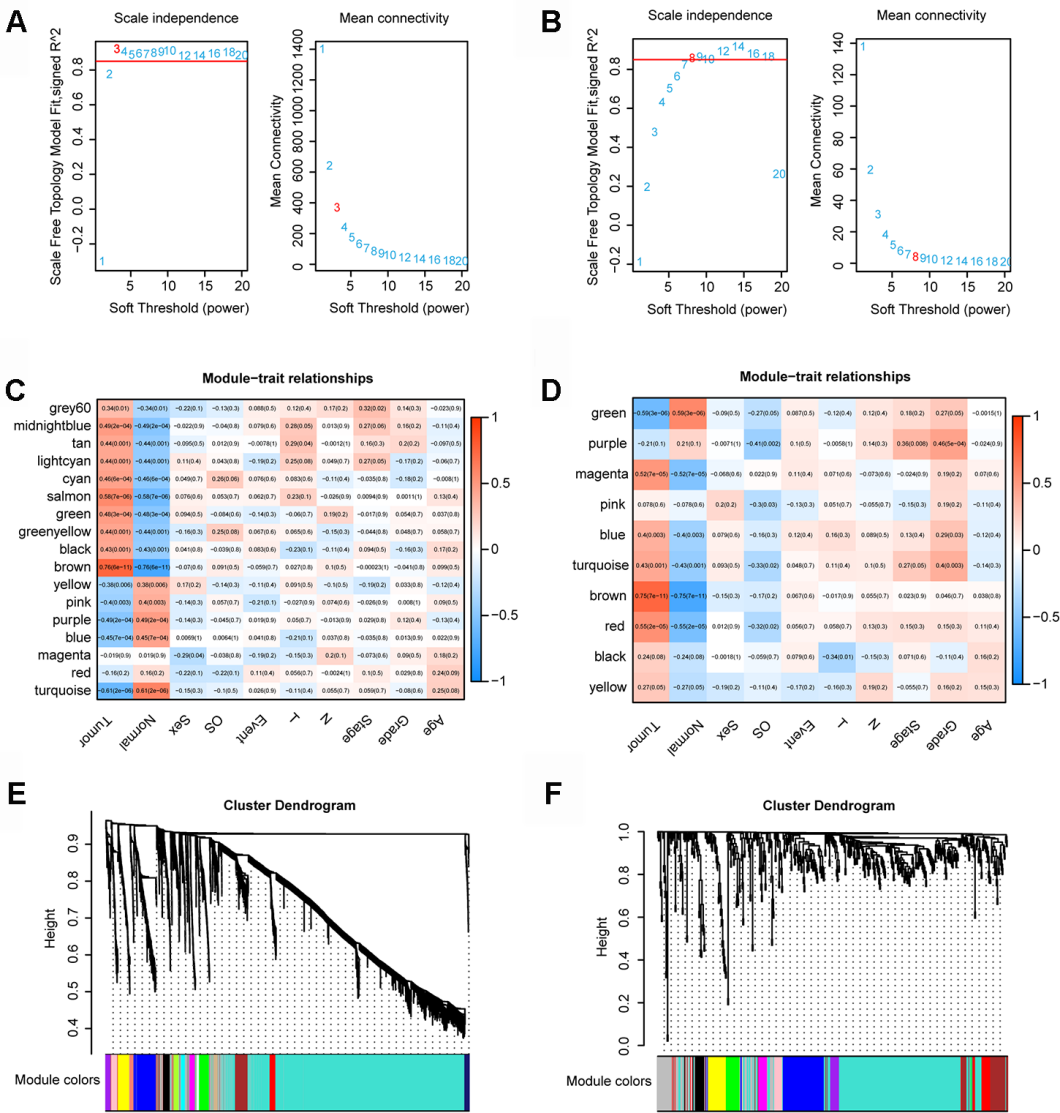
**WGCNA.** (**A**) Determination of the soft-thresholding power in the lncRNA/mRNA WGCNA. (**B**) Determination of the soft-thresholding power in the miRNAs WGCNA. (**C**) Module-trait associations of lncRNAs and mRNAs were evaluated by correlations between MEs and clinical traits. (**D**) Module-trait associations of miRNAs were evaluated by correlations between MEs and clinical traits. (**E**) Clustering dendrogram of lncRNAs and mRNAs. (**F**) Clustering dendrogram of miRNAs.

We used the expression profiles of 4721 mRNAs/lncRNAs and 486 mRNAs/lncRNAs to construct a coexpression network with the WGCNA software package in R software. In the coexpression network analysis, the β values of lncRNAs/mRNAs and miRNAs were 3 and 8, respectively (Figure 1A, and 1B). Ultimately, we obtained 17 and 10 modules in the coexpression network of lncRNAs/mRNAs and miRNAs, respectively (Figure 1C and 1D). Moreover, we calculated and plotted the relationship between each module and clinical features. Figure 1E shows a strong negative correlation between the turquoise module and tumor characteristics (module-trait weighted correlation =-0.61). However, as shown in Figure 1F, there is a significant positive correlation between the brown module and tumor characteristics (module-feature weighted correlation = 0.75).

### CeRNA network in GA

Through the prediction of online database and screening of matrix internal relationship pairs, 234 pairs of lncRNAs-miRNAs were obtained, including 55 lncRNAs (12 upregulated and 43 downregulated) and 17 miRNAs. Ninety pairs of miRNAs-mRNAs were also obtained, including 73 mRNAs (11 upregulated and 62 downregulated) (Supplementary Figure 2).

### Functional enrichment analysis

**Figure 2:**
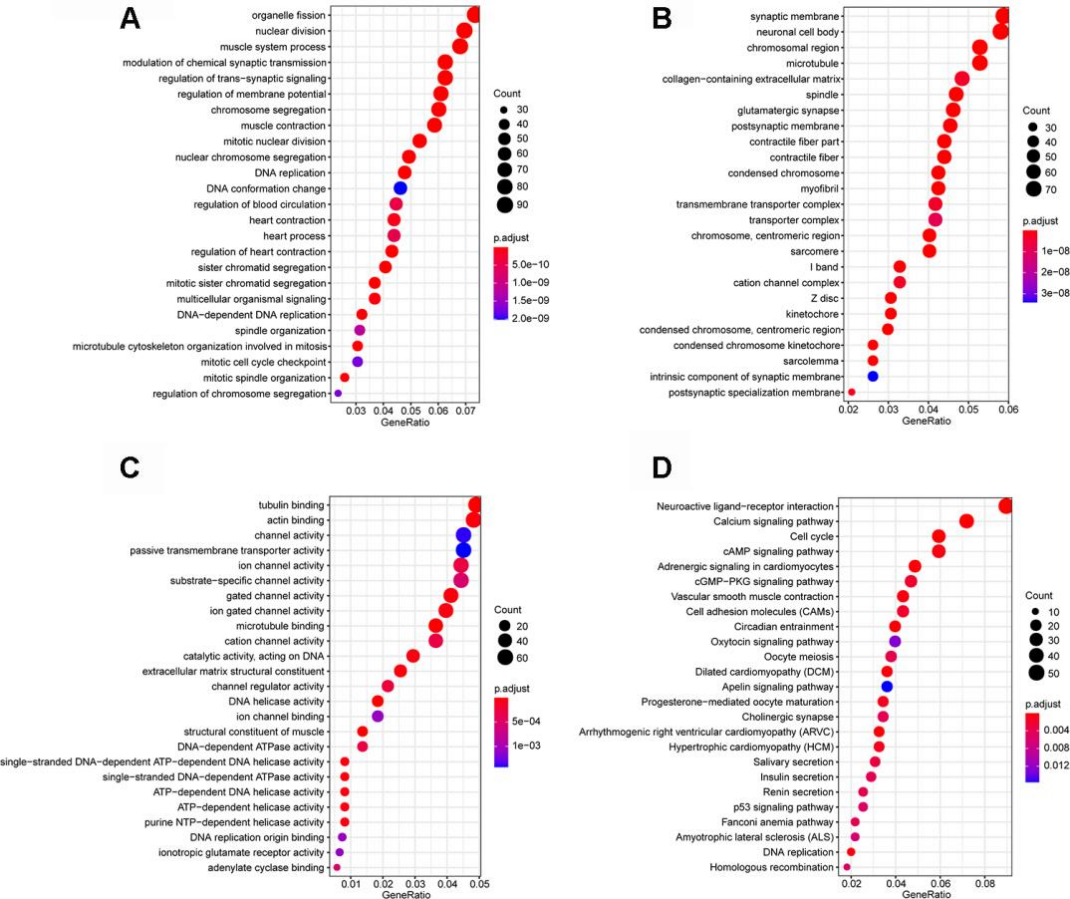
**Enrichment analyses.** (**A**) Biological process; (**B**) cellular component; (**C**) molecular function; (**D**) Kyoto Encyclopedia of Genes and Genomes (KEGG) pathways.

Functional enrichment analysis was performed to explore the Gene Ontology (GO) database terms and Kyoto Encyclopedia of Genes and Genomes (KEGG) pathways associated with genes in the turquoise module. The results indicated that the enriched biological processes mainly involved nuclear division, DNA replication, chromosome segregation, organelle fission and so on (Figure 2A). The cell components that were correlated with the resulting terms included chromosome, centromeric region, condensed chromosome, spindle and so on (Figure 2B). The results also showed that the molecular functions were related to DNA helicase activity, catalytic activity, acting on DNA, DNA-dependent ATPase activity, actin binding and so on (Figure 2C). KEGG pathway functional enrichment analysis showed that Cell cycle, p53 signaling pathway, cAMP signaling pathway, cGMP-PKG signaling pathway and DNA replication were the main pathways related to the genes in this module (Figure 2D).

### Kaplan-Meier analysis

**Figure 3:**
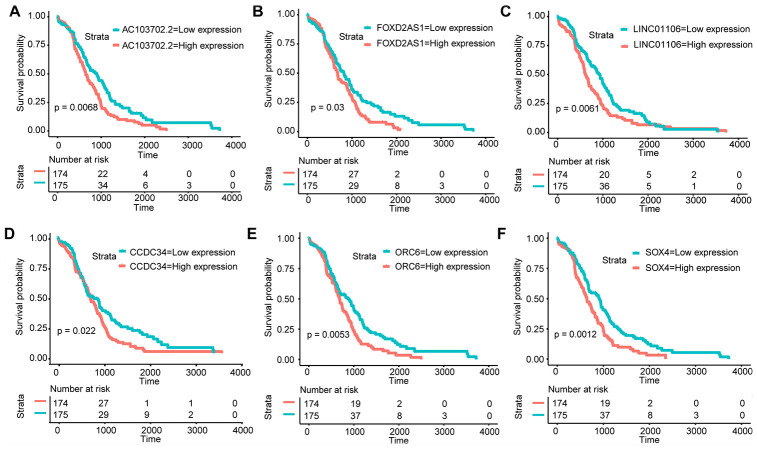
**KM analysis. KM survival curves of the hub RNAs in the ceRNA network.** (**A**) AC103702.2; (**B**) FOXD2-AS1; (**C**) LINC01106; (**D**) CCDC34; (**E**) ORC6; (**F**) SOX4.

Amo the 55 lncRNAs and 73 mRNAs, KM analysis revealed that 3 lncRNAs (LINC01106, FOXD2-AS1, and AC103702.2) and 3 mRNAs (CCDC34, ORC6, and SOX4) were identified as crucial prognostic factors. As shown in Figure 3A–3F, the survival time of GA patients with high LINC01106, FOXD2-AS1, AC103702.2, CCDC34, ORC6 and SOX4 expression was significantly shorter than that of patients with low expression. Therefore, the overexpression of LINC01106, FOXD2-AS1, AC103702.2, CCDC34, ORC6 and SOX4 may lead to a poor prognosis.

### Analysis of survival-related biomarkers in the GEPIA2 database

**Figure 4:**
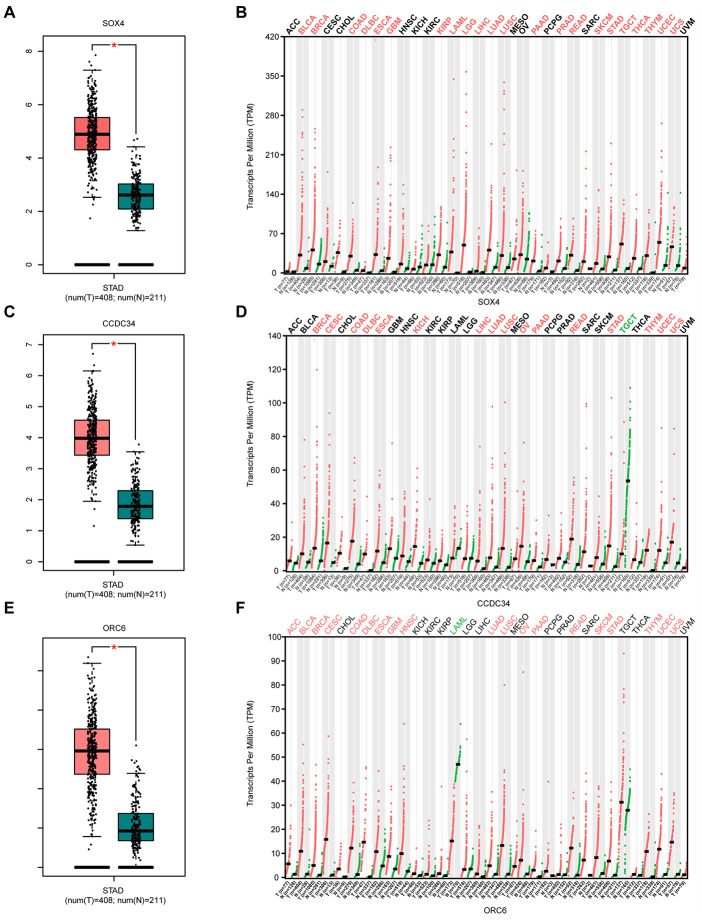
**Analysis of 3 survival-related mRNAs in the GEPIA2 database.** (**A**) Box plot of SOX4 expression in GA and normal gastric tissues. Red represents tumor tissue, while green represents normal tissue. (**B**) Dot diagram of SOX4 expression in various cancer tissues and corresponding normal tissues. Red indicates high expression, while green indicates low expression (**C**). Box plot of CCDC34 expression in GA and normal gastric tissues. (**D**) Dot diagram of CCDC34 expression in various cancer tissues and corresponding normal tissues. (**E**). Box plot of ORC6 expression in GA and normal gastric tissues. (**F**) Dot diagram of ORC6 expression in various cancer tissues and corresponding normal tissues. Abbreviations: num, Number; T, Tumor; N, Normal; ACC, Adrenocortical carcinoma; BLCA, Bladder urothelial carcinoma; BRCA, Breast invasive carcinoma; CESC, Cervical squamous cell carcinoma and endocervical adenocarcinoma; CHOL, Cholangiocarcinoma; COAD, Colon adenocarcinoma; DLBC, Diffuse large B-cell lymphoma; ESCA, Esophageal carcinoma; GBM, Glioblastoma multiforme; HNSC, Head and neck squamous cell carcinoma; KICH, Kidney chromophobe; KIRC, Kidney renal clear cell carcinoma; KIRP, Kidney renal papillary cell carcinoma; AML, Acute myeloid leukemia; LGG, Low grade glioma; LIHC, Liver hepatocellular carcinoma; LUAD, Lung adenocarcinoma; LUSC, Lung squamous cell carcinoma; MESO, Mesothelioma; OV, Ovarian serous cystadenocarcinoma; PAAD, Pancreatic adenocarcinoma; PCPG, Pheochromocytoma and paraganglioma; PRAD, Prostate adenocarcinoma; READ, Rectum adenocarcinoma; SARC, Sarcoma; SKCM, Skin Cutaneous Melanoma; STAD, Stomach adenocarcinoma; TGCT, Testicular germ cell tumors; THCA, Thyroid carcinoma; THYM, Thymoma; UCEC, Uterine corpus endometrial carcinoma; UCS, Uterine carcinosarcoma; UVM, Uveal melanoma.

Compared with normal gastric tissues, SRY-box transcription factor 4 (SOX4) is significantly overexpressed in GA tissues (Figure 4A). SOX4 also appears to be upregulated in various tumor tissues compared with corresponding normal tissues (Figure 4B). Similar situations were also obtained for CCDC34 and ORC6 (Figure 4C–4F). The three lncRNAs (LINC01106, FOXD2-AS1, and AC103702.2) also showed highly similar results (Supplementary Figure 3).

### Construction of the survival - specific ceRNA network and prognostic signature

**Figure 5:**
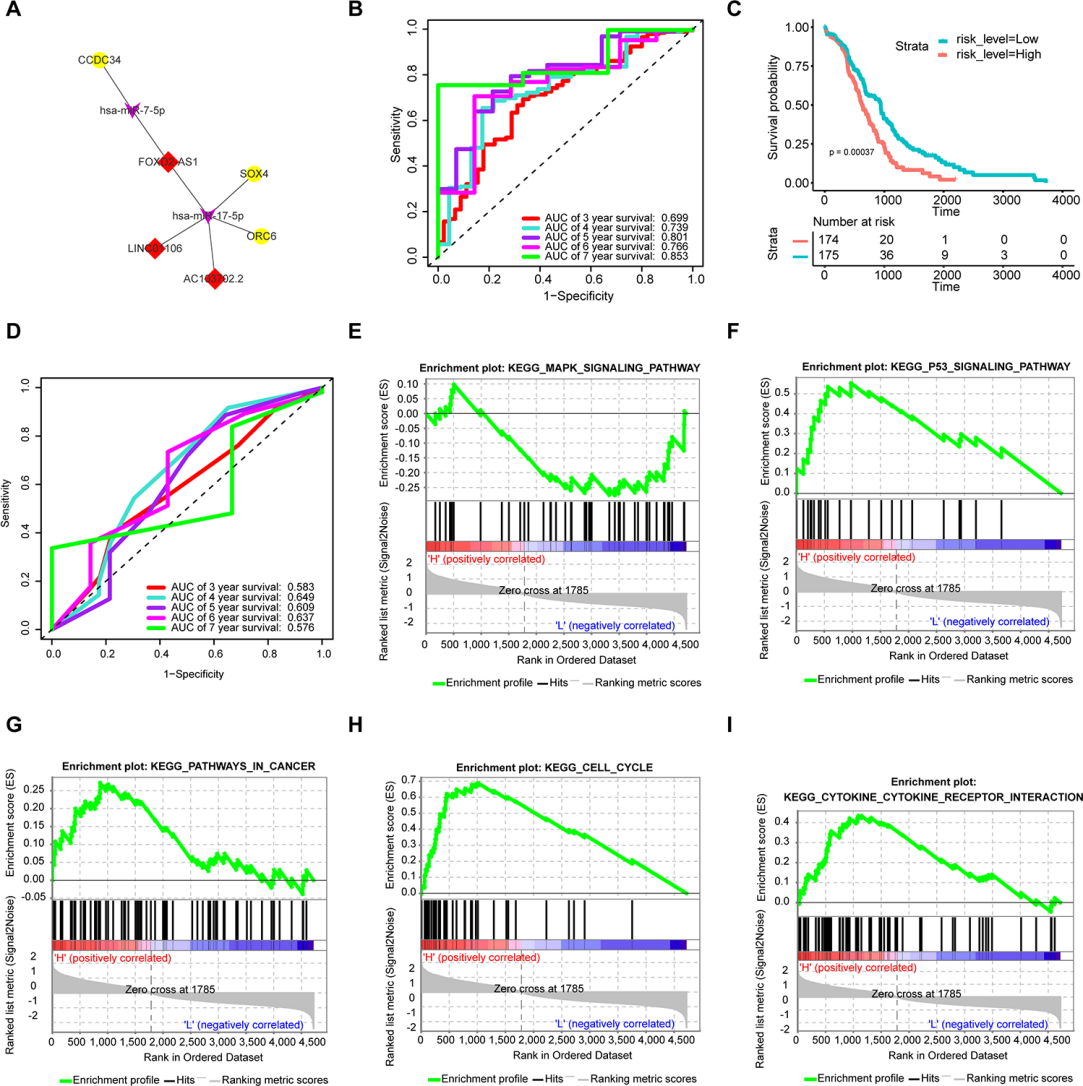
**Construction of the prognostic signature based on the survival-specific ceRNA network and GSEA.** (**A**) Hub ceRNA network. Notes: Red diamonds represent upregulated lncRNAs, purple arrows represent miRNAs, and gold circles represent upregulated mRNAs. (**B**) ROC curve analyses based on the signature. (**C**) KM curves of OS based on the signature. (**D**) ROC curve analyses based on the traditional TNM pathological staging system. (**E**–**I**) GSEA. Notes: H denotes a high signature score, while L denotes a low signature score.

As shown in Figure 5A, the upregulated lncRNAs (LINC01106, FOXD2-AS1, and AC103702.2) associate with the same upregulated mRNAs (CCDC34, ORC6, and SOX4) via hsa-miR-17-5p and hsa-miR-7-5p. To further verify the functions of the hub RNAs, we conducted multivariate Cox regression analysis and calculated the risk score. The results are presented in Table 2. All samples were randomly separated into high- and low-risk groups with the median risk score as the cut-off value. Patients in the high-risk group had significantly worse OS than those in the low-risk group (Figure 5C). In addition, receiver operating characteristic (ROC) curves were used to explore whether the prognostic ability of the ceRNA-based signature was better than that of the traditional TNM pathological staging system. The AUC values of the signature assessed for 5-year (AUC = 0.801) and 7-year (AUC = 0.853) OS were more accurate than those of the pathological stage (5-year AUC = 0.609) (Figure 5B and 5D).

**Table 2:** The results of multivariate Cox analysis.

	***β***	**HR**	**lower 95%CI**	**upper 95%CI**	***P*-value**
ORC6	0.244	1.277	1.014	1.608	0.038^*^
CCDC34	0.175	1.191	0.929	1.527	0.169
SOX4	0.588	1.801	1.377	2.355	1.71e-05^***^
LINC01106	0.256	1.292	0.899	1.857	0.167
FOXD2-AS1	0.384	1.469	1.106	1.951	0.008^**^
AC103702.2	0.440	1.553	1.143	2.111	0.005^**^

Abbreviations: *β*, coefficient; HR, Hazard ratio; *CI*, Confidence interval; ^*^P<0.05; ^**^ P<0.01; ^***^ P<0.001.

### GSEA

As shown in Figure 5E–5I, Cell cycle and P53 signaling pathway were enriched in the high-risk group. In addition, tumor-related pathways such as the MAPK signaling pathway, pathways in cancer and cytokine receptor interaction, were also enriched.

### Validation of the prognostic value of the ceRNA-based signature

**Figure 6:**
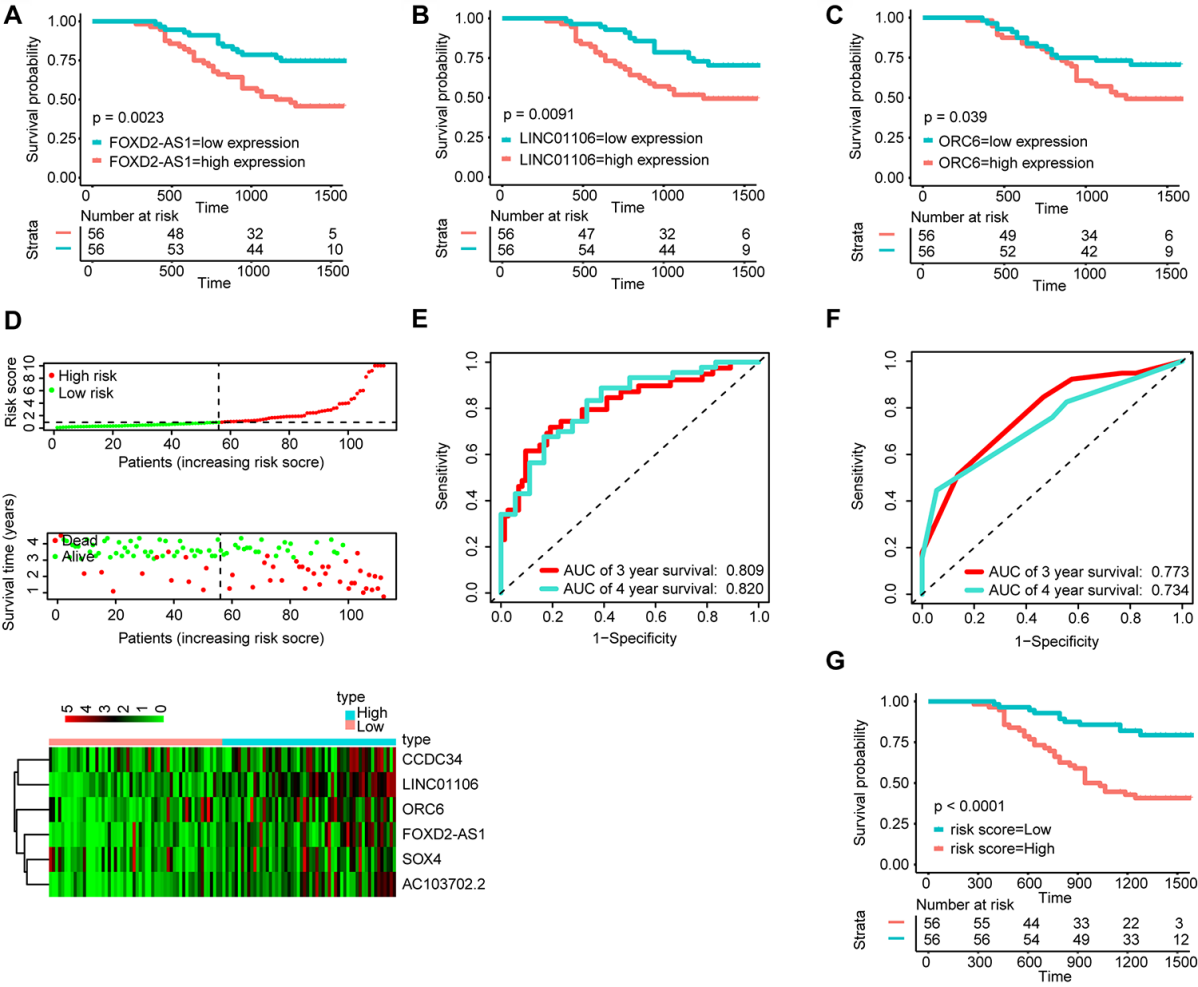
**Validation of the signature by qRT-PCR (n=112).** KM survival curves of FOXD2-AS1 (**A**), LINC01106 (**B**), and ORC6 (**C**); (**D**) Correlation between the prognostic signature and the OS of patients in the qRT-PCR cohort. Distribution of the signature scores (top), survival times (middle) and lincRNA expression levels (bottom). Black dotted lines represent the median signature score (cut-off) that was used to divide patients into the low- and high-risk groups. Red dots and lines represent patients in the high-risk group. Green dots and lines represent patients in the low-risk group. (**E**) ROC curve analyses based on the signature. (**F**) ROC curve analyses based on the traditional TNM pathological staging system. (**G**)KM curves of OS based on the signature.

To determine the stability of the nomogram, we performed a similar process in the qRT-PCR validation cohort (n = 112). First, KM analysis was performed to identify the prognostic value of FOXD2-AS1, LINC01106 and ORC6 (Figure 6A–6C). Then, with the median risk score as the cut-off point, the patients were divided into the high-risk group (n = 56) and the low-risk group (n = 56) (Figure 6D). The AUC value for 3- and 4-year OS reached 0.809 and 0.820, respectively (Figure 6E), which were still higher than the AUC values of the traditional TNM pathological staging system (Figure 6F). The KM survival curves suggested that the OS of patients in the high-risk group was significantly worse than that of patients in the low-risk grop (Figure 6G).

**Figure 7:**
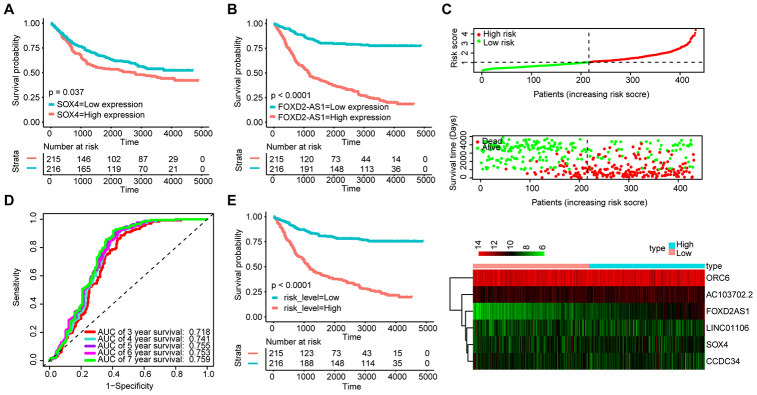
**Validation of the signature with the GSE84437 dataset (n = 431).** KM survival curves of FOXD2-AS1 (**A**) and SOX4 (**B**); (**C**) Distribution of the ceRNA-based signature scores, lncRNA expression levels and patient survival durations in the GSE84437 validation set. (**D**) ROC curve analyses based on the signature. (**E**) KM curves of OS according to the risk score.

We also validated the robustness of the signature in the GSE84437 dataset (n = 431). The KM OS curves showed that the high expression of FOXD2-AS1 and SOX4 could be related to a poor prognosis (Figure 7A and 7B). Moreover, the median risk score was used as the cut-off point to divide patients into the high-risk group (n = 215) and the low-risk group (n = 216) (Figure 7C). The AUC value for 5-year OS reached 0.755 (Figure 7D). The KM OS curves indicated that the OS of patients in the high-risk group was significantly worse than that of patients in the low-risk group (Figure 7E). These results suggest that the new signature can effectively evaluate the prognosis of GA patients.

## DISCUSSION

Despite advances in diagnosis, prognosis, and treatment, GA remains a worldwide public health concern. While some lncRNAs and mRNAs dysregulated in GA and their clinical value as potential biomarkers for prognosis have been previously reported[12, 13], this study provided additional data on two novel lncRNAs (LINC01106, and AC103702.2) and two novel mRNAs (CCDC34 and ORC6) contributing to GA and constructed a ceRNA-based signature that can be used to predict the prognosis of GA. Most importantly, the qRT-PCR validation cohort of 112 GA patients successfully verified its stability.

In the present study, we identified a turquoise module and a brown module (Brown) that were markedly associated with the GA tumor status by WGCNA. Next, we constructed a ceRNA network to identify potential prognostic lncRNA and mRNA biomarkers. The results revealed that LINC01106, FOXD2-AS1, AC103702.2, CCDC34, ORC6, and SOX4 whose high expression may indicate a poor OS. We also revealed the ceRNA relationship between these lncRNAs and mRNAs, that likely represents its mechanism of action in GA. Next, multivariate Cox regression analysis was carried out among these molecules, and the risk score was calculated. All samples were randomly separated into high- and low-risk groups with the median risk score as the cut-off value. KM analyses, ROC curves and AUC values showed that the signature based on the ceRNAs objectively and accurately predicted the prognosis of patients with GA. The results were successfully verified in the qRT-PCR validation cohort and the GSE84437 dataset.

LINC01106 is broadly distributed in the brain (RPKM 31.9), stomach (RPKM7.0) and 17 other tissues. Sun et al. [14] found that LINC01106 was overexpressed in colon cancer, and survival analysis showed that LINC01106 was strongly associated with the OS of colorectal cancer patients. Similarly, we found that LINC01106 was overexpressed in GA tissue and associated with a poor prognosis.

The protein encoded by ORC6 is a subunit of the ORC complex, which includes a core complex consisting of ORC2, ORC3, ORC4, and ORC5, loosely interacting with ORC6. Gene silencing studies with siRNAs demonstrated that this protein plays an essential role in coordinating chromosome replication and segregation with cytokinesis [15]. Research has also shown that the expression of ORC6 in colon cancer tissues is high and associated with invasion depth [16]. Moreover, a reduction in ORC6 expression sensitizes human colon cancer cells to 5-fluorouracil and cisplatin [17]. Our results suggest that ORC6 overexpression in GA likely causes poor OS.

Coiled-coil domain containing 34 (CCDC34), also known as renal carcinoma antigen NY-REN-41, is a protein-coding and disease related gene. Current research have shown that CCDC34 is upregulated in a variety of tumors and contributes to the malignant behaviors of cancer cells [18–22]. Recently, Gong et al. [19] found that CCDC34 was overexpressed in bladder cancer and promoted cell proliferation and migration. Lin et al. [21] found that it was also upregulated in hepatocellular carcinoma and contributes to cell proliferation and metastasis. However, its expression and role in GA have not been reported. In our study, we found that CDCC34 was highly expressed in GA tissues and not conducive to prognosis. However, little is known about its specific role in GA. Current studies have confirmed that SOX4 and FOXD2-AS1 are overexpressed in GA tissues and promote the proliferation and migration of GA cells [23–25]. These results further validate the authenticity of the current study. In addition, by constructing a ceRNA network, we also found that FOXD2-AS1 may increase the expression of SOX4, ORC6 and CCDC34 through hsa-miR-17-5p and hsa-miR-7-5p, respectively, and thus play a regulatory role in the growth, differentiation and migration of GA cells. In turn, SOX4 may be increased by LINC01106, FOXD2-AS1 and AC103702.2 through hsa-miR-17-5p, and activate the biological behavior of GA.

To validate our conclusion, we conducted qRT-PCR experiments to demonstrate that LINC01106, FOXD2-AS1, AC103702.2, CCDC34, ORC6, and SOX4 are differentially expressed in GA and corresponding normal tissues. Our results showed that the expression of LINC01106, FOXD2-AS1, AC103702.2, CCDC34, ORC6, and SOX4 was higher in GA tissues than in corresponding normal tissues (Supplementary Figure 4). We also found that SOX4, ORC6, and CCDC34 were more highly expressed in adenocarcinoma tissue than in normal gastric tissue using Western blot (WB) assays and immunohistochemistry (IHC). Moreover, SOX4, ORC6, and CCDC34 are located mainly in the nucleus of adenocarcinoma tissues (Supplementary Figure 5). It is worth noting that the lncRNA-miRNA relationship pair were constructed based on the correlation of the internal expression of the matrix and not the results of the analysis of the online databases miRcode and starBase, because very few relationship pairs remained.

In summary, we identified two novel lncRNAs (LINC01106 and AC103702.2) and two novel mRNAs (ORC6 and CCDC34) related to the prognosis of GA. We also constructed a survival-specific ceRNA network between these lncRNAs and mRNAs, and developed a prognostic signature for GA, that shows better accuracy than the traditional TNM pathological staging system. Our research will help further understand the molecular mechanism of GA and provide new insights for the treatment and prognosis of GA.

## MATERIALS AND METHODS

### Data acquisition and preprocessing

Open data sets were downloaded from the TCGA (https://portal.gdc.cancer.gov/) and GEO (https://www.ncbi.nlm.nih.gov/gds/) databases, including the lncRNA, mRNA and miRNA expression profiles of GA specimens and the corresponding clinical follow-up data. The RNA expression profile was downloaded in fragments per kilobase million (FPKM) format. The data used in this study met the following criteria: (1) RNAs with nonzero expression levels (accounting for 75% of all samples); (2) the median and SD of the RNAs were larger than 1.2, and (3) exact follow-up times.

### Differential expression analysis

Differentially expressed RNAs were identified by the edgeR package in R software [26]. Significantly expressed RNAs were identified by setting the adjusted *P* value to < 0.05 and the |log_2_FC (fold change) | > 1(|log_2_FC > 1| and adjusted *FDR* < 0.05).

### Construction of the weighted gene coexpression network

The WGCNA package implemented in R software [27] was used to build a gene coexpression network based on the gene, lncRNA and miRNA expression characteristics. A scale-free plot was used to evaluate whether the network exhibited scale-free topology. The power value of the soft threshold of the adjacency matrix met the scale-free topology criterion. On this basis, we built a scale-free network and topological overlap matrix (TOM). The dynamic tree cutting method was used to generate modules with the following main parameters: deepSplit of 2 and min module size of 30 (for miRNAs, the min module size was set as 10). The height cut-off was set to 0.25, and if the module's similarity was > 0.8, the modules were merged. Based on Pearson's tests, we further determined the association between module eigengenes (MEs) and external clinical information, including sample status. If the P-value was < 0.05 and the correlation coefficient was>0.9, it was considered a significant correlation.

### CeRNA network construction and analysis

According to the results of the WGCNA, we selected all the mRNAs/lncRNAs in the most negative correlation module and the miRNAs in the most positive correlation module to build the ceRNA network. In short, the related ceRNA network in GA was constructed through four stages: First, we identified the internal network relationship based on the lncRNA/mRNA and miRNA expression matrices via the WGCNA package in R software, in which the weight coefficient was controlled to be greater than 0.15. Second, miRTarBase (http://mirtarbase.cuhk.edu.cn/)[28], TargetScan (www.targetscan.org/), miRDB (mirdb.org/)[29] and miRWalk (http://mirwalk.umm.uni-heidelberg.de/)[30] were used to predict the target genes of the miRNAs. Then, to further ensure the reliability of the constructed ceRNA network, we compared the predicted relationship pairs with the internal network, and we retained only the data of overlapping interaction pairs for further analysis. Finally, Cytoscape 3.7.2 software was used to construct and visualize the ceRNA network based upon the remaining interaction pairs.

### Module function annotation

GO and KEGG analyses were realized through the org.Hs.eg.db package and clusterProfiler in R software. GO consists of three terms: biological process (BP), molecular function (MF), and cellular composition (CC). All important GO terms and KEGG pathways were filtered according to a P < 0.05 and at least two associated mRNAs.

### Survival analysis

KM survival analysis was performed to evaluate the association between disease prognosis and lncRNAs and mRNAs. We also analyzed the clinical data of 349 GA patients with a nonzero survival time from the TCGA database and drew the survival curves of all lncRNAs and mRNAs in the ceRNA network using the R package “survival”. When the *P*-value of lncRNA or mRNA was < 0.05, it was considered statistically significant and indicated that the lncRNA or mRNA has potential prognostic value.

### Analysis of survival-related biomarkers in the GEPIA2 database

GEPIA2 is an updated version of GEPIA that can be used to analyze the RNA sequencing expression data of 9736 tumor samples and 8587 normal samples from the TCGA and the Genotype-Tissue Expression (GTEx) projects, using a standard processing pipeline [31]. We used this online tool to analyze the expression levels of six survival-related biomarkers in various organs and tissues based on the TCGA and GTEx databases. The differential method was based on the “limma” R package, and we used log2 (TPM + 1) for log-scale. The |log2FC| cut-off was set as 1, while the q-value cutoff was set as 0.05. We also drew a box plot of the expression levels of the survival-related biomarkers in GA tissue via internal tools.

### Construction of the survival-specific ceRNA network and prognostic signature

We obtained the connections between 3 lncRNAs (LINC01106, FOXD2-AS1, and AC103702.2) and 3 mRNAs (CCDC34, ORC6, and SOX4), whose *P*-value was < 0.05 in the survival analysis, from the lncRNA-miRNA and mRNA-miRNA relationship pairs. The results were visualized using Cytoscape 3.7.2 software. Next, multivariate Cox regression was used to identify the corresponding coefficients of the GA prognostic signature and to calculate the risk score (risk score = expGene1 ×βGene1 + expGene2 × βGene2 + expGenen × βGenen (exp, prognostic gene expression level; β, multivariate Cox regression model regression coefficients)) by using the R packages “glmnet”, “survminer” and “survival”. All samples were randomly separated into high- and low-risk groups with the median risk score as the cut-off value. Survival for each group was evaluated by the KM analysis and the log-rank test. The ROC curve and AUC were drawn with the R package “timeROC”.

### qRT-PCR

Total RNA was dreverse transcribed into cDNA with random primers using a Transcriptor First Strand cDNA Synthesis Kit (Roche, Penzberg, Germany) following the manufacturer’s instructions. The expression levels of the RNAs were measured by qRT-PCR using FastStart Essential DNA Green Master Mix (Roche, Penzberg, Germany) on a Roche LightCycler 480 (Roche, Penzberg, Germany). RNA expression was normalized to that of GAPDH. All quantitative PCRs were conducted in triplicate. Divergent primers, rather than the more commonly used convergent primers, were designed for the RNAs. We verified the specificity of the PCR primers using BLAST. A single peak in the melting curve indicated that the PCR products were specific. The primers used in the study are presented in Supplementary Table 1.

## Supplementary Material

Supplementary Material

Supplementary Figures

Supplementary Table 1
