# Supplementary material for: Development and validation of a novel prognostic signature in gastric adenocarcinoma
Source: Aging (Albany NY). 2020 Nov 8;12(21):22233–52. doi: 10.18632/aging.104161 (PMC11623975; doi:10.18632/aging.104161)
Supplement: Supplementary Material [file aging-12-104161-s001.pdf]

SUPPLEMENTARY MATERIALS

SUPPLEMENTARY METHODS

Immunohistochemistry

We performed immunohistochemistry (IHC) as described in our previous studies [1]. Briefly, sections 4-μm thick were subjected to deparaffinization, antigen retrieval, and blockage of non-specific binding, with the blockage performed by incubation with 10% normal goat serum for 15 min. The sections were incubated with primary antibodies for ORC6 (1:100, Proteintech), CCDC34 (1:100, invitrogen), and SOX4 (1:100, Abcam) at 4°C overnight, and then with a biotinylated secondary antibody. Subsequently, slides were stained with 3,3-diaminobenzidine tetrahydrochloride.

Western blot analysis

Total protein was extracted from tissues using rotor and radio immunoprecipitation assay (RIPA) lysis buffer. Equal amounts of protein were separated by SDS-PAGE in a 12% gel and transferred to a nitrocellulose membrane. The proteins were detected using an enhanced chemiluminescence system according to the manufacturer's instructions. Membranes were incubated overnight with the following primary antibodies: anti-ORC6 (Proteintech), anti-SOX4 (Abcam), anti-CCDC34 (invitrogen).

REFERENCES

1. Zhang T, Wang Z, Liu Y, Huo Y, Liu H, Xu C, Mao R, Zhu Y, Liu L, Wei D, Liu G, Pan B, Tang Y, et al. Plastin 1 drives metastasis of colorectal cancer through the IQGAP1/Rac1/ERK pathway. Cancer Sci. 2020; 111:2861–71. https://doi.org/10.1111/cas.14438 PMID:32350953
