# Supplementary material for: Development and validation of a novel prognostic signature in gastric adenocarcinoma
Source: Aging (Albany NY). 2020 Nov 8;12(21):22233–52. doi: 10.18632/aging.104161 (PMC11623975; doi:10.18632/aging.104161)
Supplement: Supplementary Figures [file aging-12-104161-s002.pdf]

SUPPLEMENTARY FIGURES

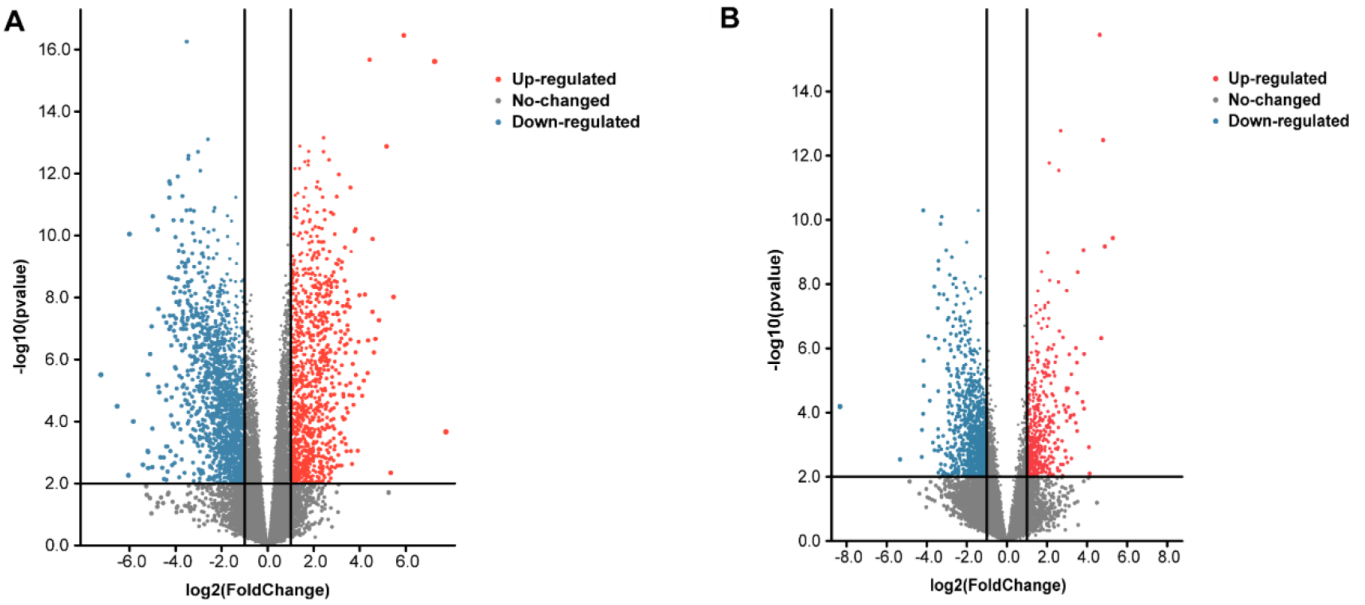

Supplementary Figure 1. Differentially expressed mRNAs and lncRNAs between stomach adenocarcinoma and para-carcinoma tissues. (**A**) Volcano plot of the differentially expressed mRNAs. (**B**) Volcano plot of the differentially expressed lncRNAs. Red indicates high expression and blue indicates low expression.

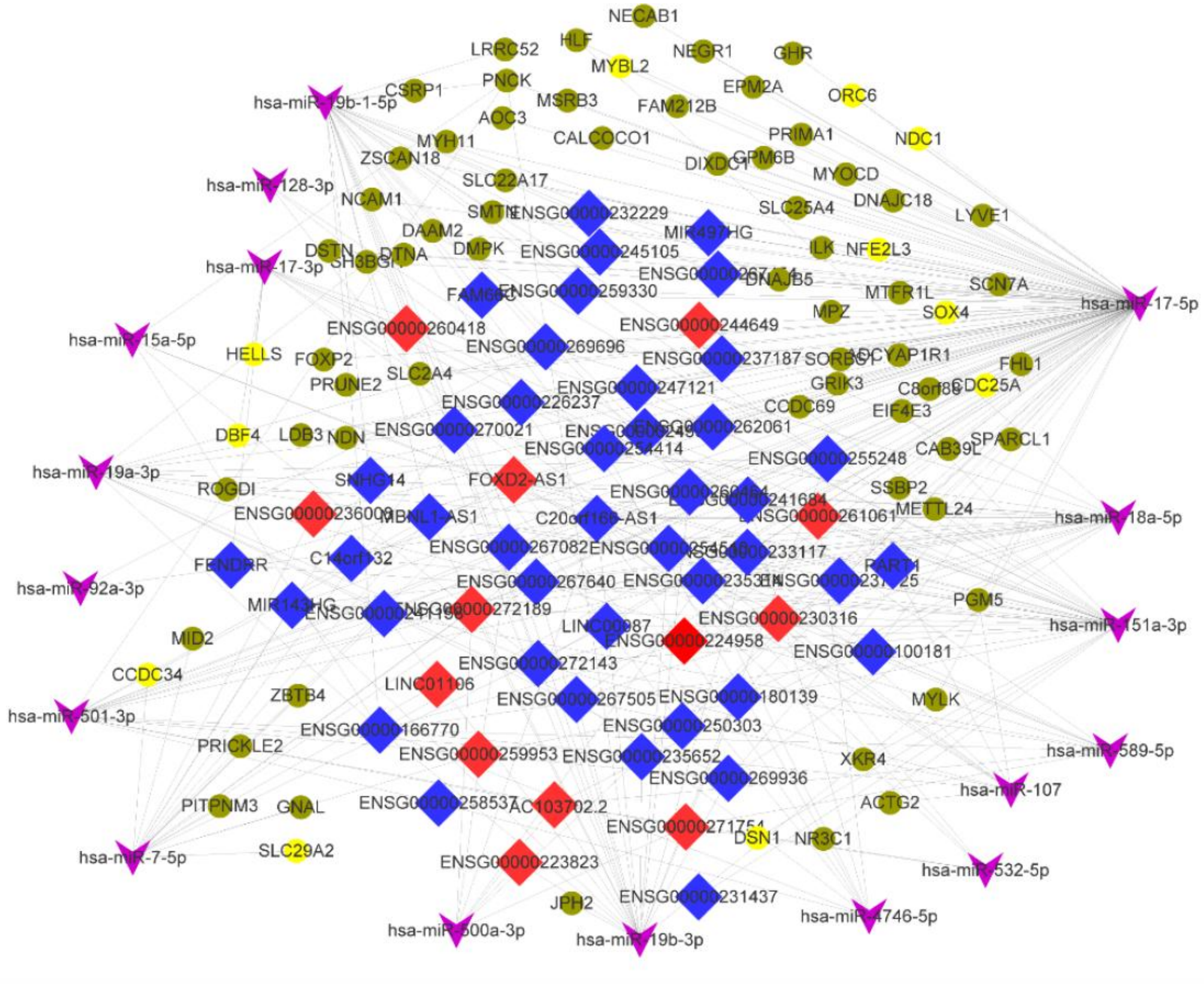

Supplementary Figure 2. CeRNA network. Turquoise module of lncRNA and mRNA and Brown module of miRNA ceRNA network. Notes: Red diamonds represent upregulated lncRNA, while blue diamonds represent downregulated lncRNA, Purple arrow shapes represent miRNA, and golden rounds represent upregulated mRNA, while Brown yellow rounds represent downregulated mRNA.

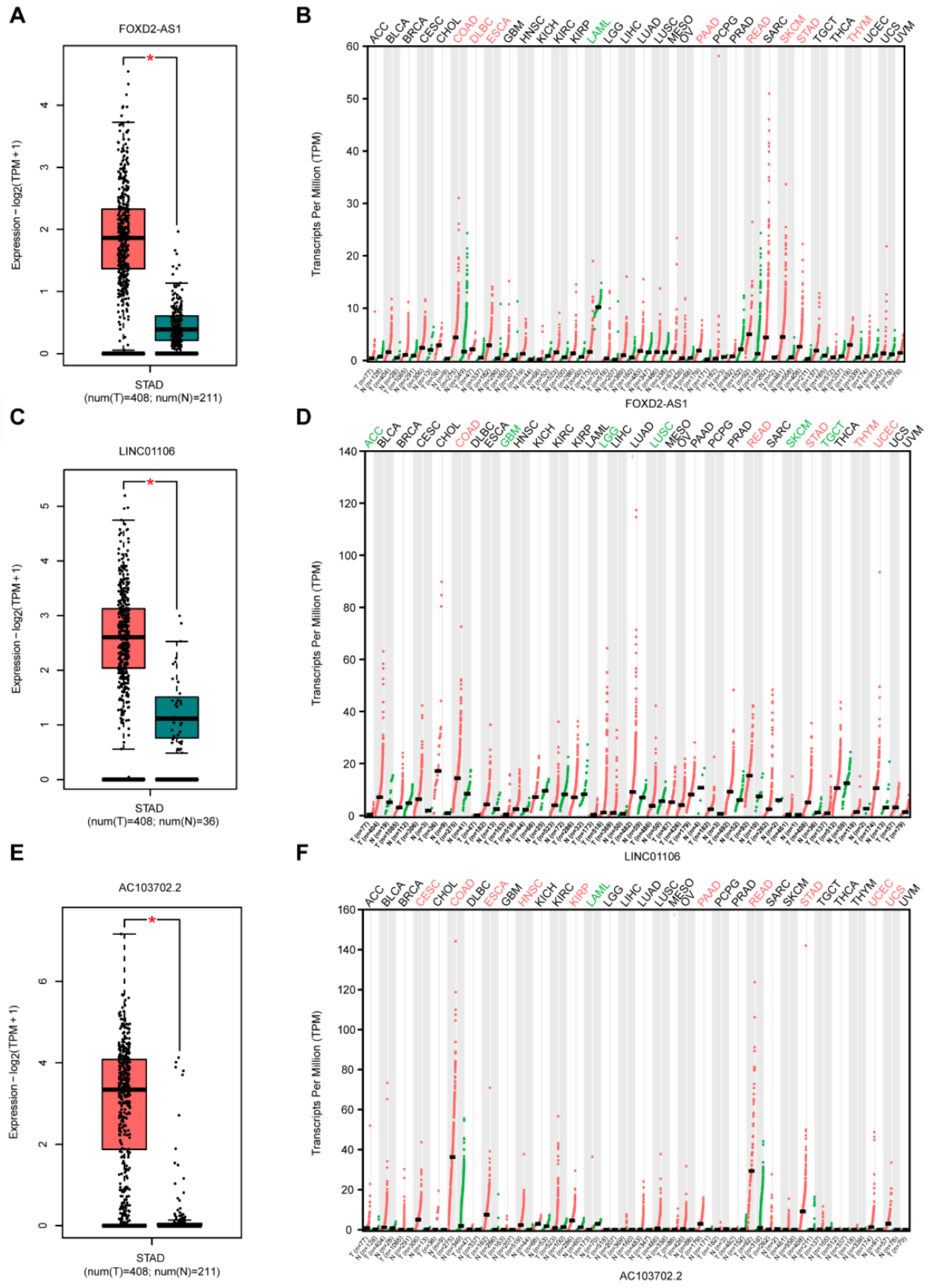

Supplementary Figure 3. Analysis of 3 survival-related lmcRNAs in the GEPIA2 database. (**A**) Box plot of FOXD2-AS1 expression in GA and normal gastric tissues. Red represents tumor tissue, while green represents normal tissue. (**B**) Dot diagram of FOXD2-AS1 expression in various cancer tissues and corresponding normal tissues. Red indicates high expression, while green indicates low expression (**C**). Box plot of LINC01106 expression in GA and normal gastric tissues. (**D**) Dot diagram of LINC01106 expression in various cancer tissues and corresponding normal tissues. (**E**) Box plot of AC103702.2 expression in GA and normal gastric tissues. (**F**) Dot diagram of AC103702.2 expression in various cancer tissues and corresponding normal tissues. Abbreviations: num, Number; T, Tumor; N, Normal; ACC, Adrenocortical carcinoma; BLCA, Bladder urothelial carcinoma; BRCA, Breast invasive carcinoma; CESC, Cervical squamous cell carcinoma and endocervical adenocarcinoma; CHOL, Cholangiocarcinoma; COAD, Colon adenocarcinoma; DLBC, Diffuse large B-cell lymphoma; ESCA, Esophageal carcinoma; GBM, Glioblastoma multiforme; HNSC, Head and neck squamous cell carcinoma; KICH, Kidney chromophobe; KIRC, Kidney renal clear cell carcinoma; KIRP, Kidney renal papillary cell carcinoma; AML, Acute myeloid leukemia; LGG, Low grade glioma; LIHC, Liver hepatocellular carcinoma; LUAD, Lung adenocarcinoma; LUSC, Lung squamous cell carcinoma; MESO, Mesothelioma; OV, Ovarian serous cystadenocarcinoma; PAAD, Pancreatic adenocarcinoma; PCPG, Pheochromocytoma and paraganglioma; PRAD, Prostate adenocarcinoma; READ, Rectum adenocarcinoma; SARC, Sarcoma; SKCM, Skin Cutaneous Melanoma; STAD, Stomach adenocarcinoma; TGCT, Testicular germ cell tumors; THCA, Thyroid carcinoma; THYM, Thymoma; UCEC, Uterine corpus endometrial carcinoma; UCS, Uterine carcinosarcoma; UVM, Uveal melanoma.

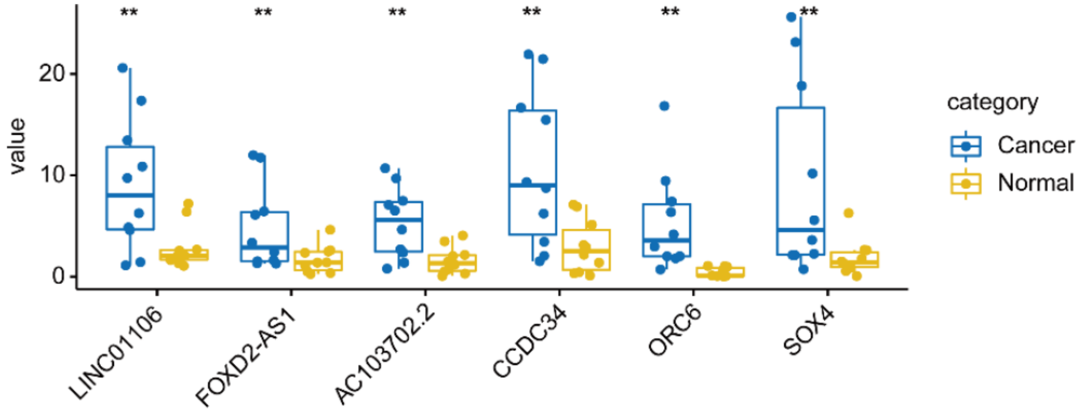

Supplementary Figure 4. The expression of LINC01106, FOXD2-AS1, AC103702.2, CCDC34, ORC6, and SOX4 in 10 pairs of gastric adenocarcinoma tissues and corresponding normal tissues by qRT-PCR; **, P < 0.01.

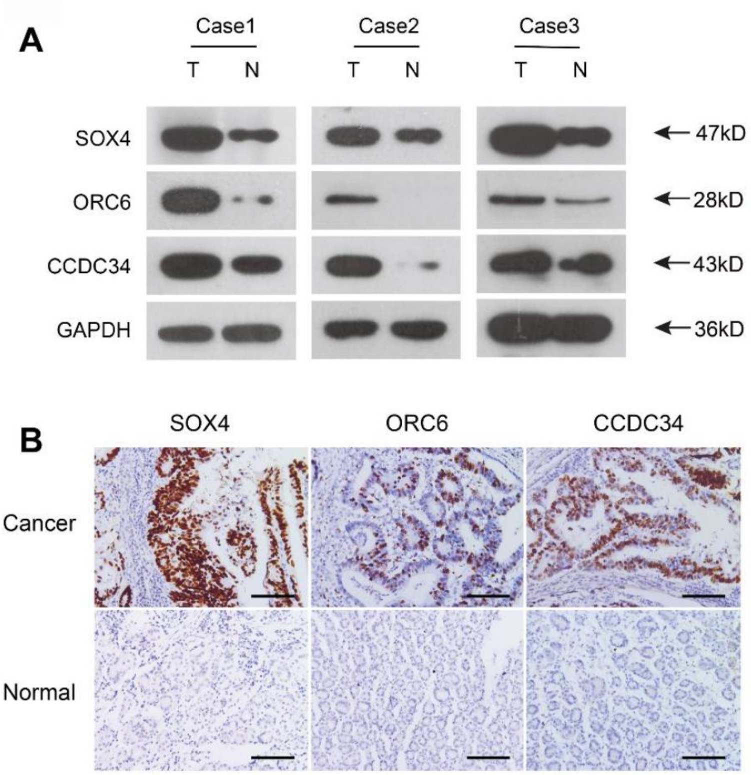

Supplementary Figure 5. The protein expression of SOX4, ORC6, and CCDC34 was analyzed in adenocarcinoma tissue and normal gastric tissue using Western blot assays (**A**) and immunohistochemistry (**B**).
