# Supplementary material for: Development and validation of a novel prognostic signature in gastric adenocarcinoma
Source: Aging (Albany NY). 2020 Nov 8;12(21):22233–52. doi: 10.18632/aging.104161 (PMC11623975; doi:10.18632/aging.104161)
Supplement: Supplementary Table 1 [file aging-12-104161-s003.pdf]

SUPPLEMENTARY TABLE

Supplementary Table 1. Primers used in the study.

Gene	Sequence
FOXD2-AS1	5’- AAGCGATCAGCTCCCTTAGC-3’<br>3’- CAGACGCGTGGTGGTTATCT-5’
AC103702.2	5’- TACACTGCCTCCTCTCACCAACC-3’<br>3’- ACTTCTCCCCACTCCCTTTCTTCC-5’
LINC01106	5’- CTGTGTCGGTGAGTTCTGGTCAAC-3’<br>3’- TCCATTCTCCTCTCCCGTGTAAGC-5’
SOX4	5’- AAGATCATGGAGCAGTCGCC-3’<br>3’- CGCCTCTCGAATGAAAGGGA-5’
CCDC34	5’- GGTAGCCAGCCACAACTGTCATC-3’<br>3’- TTAGAGACGCCCGCCACTACG-5’
ORC6	5’- TGGAGGCTAAGTCTGGGCAGTG-3’<br>3’- GTGCTGGGATTACAGGCGTGAG-5’
GAPDH	5’-GAAAGCCTGCCGGTGACTAA-3’<br>3’-GCCCAATACGACCAAATCAGAG-5’
